# New crosstalk between probiotics *Lactobacillus plantarum* and *Bacillus subtilis*

**DOI:** 10.1038/s41598-019-49688-8

**Published:** 2019-09-11

**Authors:** Tao Yu, Jian Kong, Li Zhang, Xinyi Gu, Mingyu Wang, Tingting Guo

**Affiliations:** 1 Qilu Hospital, Shandong University, No. 44 Wenhuaxi Road, Jinan, 250012 P.R. China; 2 State Key Laboratory of Microbial Technology, Shandong University, No. 72 Binhai Road, Qingdao, 266237 P.R. China

**Keywords:** Symbiosis, Applied microbiology

## Abstract

It was reported that oral administration of *Bacillus* favored the growth of *Lactobacillus* in the intestinal tract. Here, this phenomenon was confirmed by co-cultivation of *Bacillus subtilis* 168 and *Lactobacillus plantarum* SDMCC050204-pL157 *in vitro*. To explain the possible molecular mechanisms, *B*. *subtilis* 168 cells were incubated in simulated intestinal fluid at 37 °C for 24 h, and up to 90% of cells autolysed in the presence of bile salts. Addition of the autolysate to medium inoculated with *Lb*. *plantarum* SDMCC050204 decreased the concentration of H_2_O_2_ in the culture, alleviated DNA damage and increased the survival of *Lb*. *plantarum*, as like the results of exogenous heme addition. These results suggested that the autolysate provided heme, which activated the heme-dependent catalase KatA in *Lb*. *plantarum* SDMCC050204. HPLC confirmed the presence of heme in the autolysate. Disruption of the *Lb*. *plantarum* SDMCC050204 *katA* gene abolished the protective effect of the *B*. *subtilis* 168 autolysate against H_2_O_2_ stress. We thus hypothesized that the beneficial effect of *Bacillus* toward *Lactobacillus* was established through activation of the heme-dependent catalase and remission of the damage of reactive oxygen species against *Lactobacillus*. This study raised new crosstalk between the two frequently-used probiotics, highlighting heme-dependent catalase as the key mediator.

## Introduction

Numerous microorganisms that inhabit the human gastrointestinal tract form a dynamic and diverse community, referred to as the gut microbiota^1^. An ever-increasing number of studies have pointed out the relationship between gut microbiota and body health, showing that these microbial populations impact an array of physiological functions in the gastrointestinal tract, including digestion, immune response and disease prevention^2–4^. *Bacillus* and *Lactobacillus* strains are bacterial members of the gut microbiota and are widely used as probiotics^1,5–7^. In China, *B*. *subtilis*, *B*. *licheniformis* and *B*. *cereus* are ingested by adults, infants and newborns to relieve diarrhea and build health intestinal microbiota. *Bacillus* strains are considered to efficiently antagonize pathogenic bacteria, while promote the growth of *Lactobacillus* to balance the microbiota^8^, achieving therapeutic purposes. The question is then, how does *Bacillus* crosstalk with *Lactobacillus*?

Previously, it was reported that co-cultivation with *Bacillus* strains could facilitate the growth of *Lactobacillus* in animal intestinal tracts^9–12^. This beneficial effect may be interpreted by the “Biological Oxygen-Capturing Theory”. Briefly, according to this theory, *Bacillus* spores are orally administered because of their resistance to the gastrointestinal tract. When arriving in the small intestine, where is rich in nutrients, the spores are induced to germinate, proliferate, and then resporulate^13^. This process consumes oxygen in the intestinal tract, and generates an anaerobic environment for the proliferation of *Lactobacillus*^14^. Moreover, *Bacillus* strains produce bioactive molecules including hydrolytic enzymes (amylase and protease), antioxidative enzymes (catalase and superoxide dismutase) and surface proteins that could favor the growth and viability of *Lactobacillus*^15,16^.

Normally, lactic acid bacteria are sensitive to reactive oxygen species (ROS) because they do not equip with perfect antioxidant systems found in aerobic microorganisms^17^. In fact, several *Lactobacillus* strains, including *Lb*. *plantarum*, *Lb*. *brevis* and *Lb*. *casei*, have the heme-dependent catalase coding gene in their genomes^18–20^. Catalase catalyzes the decomposition of the ROS hydrogen peroxide. However, owing to their lack of the heme biosynthesis ability, these *Lactobacillus* strains are defined as catalase-negative strains. When these *Lactobacillus* cells are supplemented with exogenous heme, the heme-dependent catalase in these cells could be activated, which increase survival and biomass of the *Lactobacillus* strains^20^. However, whether *Bacillus* is involved in this process in the intestinal tract is yet to be uncovered besides the “Biological Oxygen-Capturing Theory”.

In this study, we performed co-cultivation of *B*. *subtilis* 168 and *Lb*. *plantarum* SDMCC050204-pL157, analyzed the presence of heme in autolysate of *B*. *subtilis* 168, and confirmed the role of the autolysate in activation of catalase (KatA) in *Lb*.*plantarum* SDMCC050204. The aim of this study was to propose a new crosstalk model between *Bacillus* and *Lactobacillus* strains in the intestinal tract after oral administration of *Bacillus*.

## Materials and Methods

### Bacterial strains, culture media, and growth conditions

Bacterial strains and plasmids used in this study are listed in Table 1. *B*. *subtilis* 168 and *Escherichia coli* DH5α were grown aerobically in Luria-Bertani (LB) medium at 37 °C. *Lb*. *plantarum* strains were grown in de Man, Rogosa and Sharpe (MRS) broth containing 0.5% glucose at 37 °C in two different conditions, either (i) static cultivation in 100-mL Erlenmeyer flasks containing 50 mL medium, or (ii) aerated cultivation in 300-mL Erlenmeyer flasks containing 50 mL medium with agitation on a rotary shaker at 200 rpm. When appropriate, the following antibiotics were added to the medium: ampicillin (100 μg/mL for *E*. *coli*), chloramphenicol (5 μg/mL for *Lb*. *plantarum*) and erythromycin (5 μg/mL for *Lb*. *plantarum*). When required, 20 μM heme (Sigma, USA) was added to medium. Cell turbidity was monitored by the optical density at 600 nm (OD_600_).

**Table 1:** Bacterial strains and plasmids used in this study.

Strain, plasmid or primer	Feature(s) or sequence	Reference
**Strain**		
*B*. *subtilis* 168	Standard strain	CGMCC
*Lb*. *plantarum* SDMCC050204	Wild-type strain isolated from Chinese artisanal cheese; Whole genome sequencing (unpublished data)	Our laboratory
*Lb*. *plantarum* SDMCC050276	Wild-type strain isolated from silage	Our laboratory
*Lb*. *plantarum* SDMCC050277	Wild-type strain isolated from silage	Our laboratory
*Lb*. *plantarum* SDMCC050204-pL157	*Lb*. *plantarum* SDMCC050204 containing the plasmid pL157; Cm^r^	This work
*Lb*. *plantarum* SDMCC050276-pL157	*Lb*. *plantarum* SDMCC050276 containing the plasmid pL157; Cm^r^	This work
*Lb*. *plantarum* SDMCC050277-pL157	*Lb*. *plantarum* SDMCC050277 containing the plasmid pL157; Cm^r^	This work
*Lb*. *plantarum* SDMCC050204ΔkatA	*Lb*. *plantarum* SDMCC050204 with the heme-dependent catalase gene (*katA*) inactivation in the genome	This work
*E*. *coli* DH5α	Cloning host	Novagen
**Plasmid**		
pL157	Cm^r^; shuttle vector in lactobacilli and *E*. *coli*	^21^
pUC-erm	Amp^r^, Erm^r^; the erythromycin resistance gene from the vector pMG36e was cloned into the BamHI site of the vector pUC19	^27^

### Co-cultivation of *Lb*. *plantarum* SDMCC050204-pL157 with *B*. *subtilis* 168

To easily distinguish and enumerate *Lb*. *plantarum* easily in co-culture with *B*. *subtilis* 168 (which is chloramphenicol sensitive), *Lb*. *plantarum* SDMCC050204 was conferred with a chloramphenicol resistance phenotype by transformation of plasmid pL157 by electroporation^21,22^, generating *Lb*. *plantarum* SDMCC050204-pL157. Since previous report showed the physical stability of pL157 in host cells without selective pressure^21^, chloramphenicol was not added into the co-culture of *Lb*. *plantarum* SDMCC050204-pL157 and *B*. *subtilis* 168.

*Lb*. *plantarum* SDMCC050204-pL157 and *B*. *subtilis* 168 were individually cultivated to reach early stationary phase. The cells were collected by centrifugation at 5,000 × *g* for 5 min, washed three times with sterile saline solution and resuspended in MRS broth. Then, 5.6 × 10^8^ CFU/mL of *Lb*. *plantarum* SDMCC050204-pL157 and 6.0 × 10^7^ CFU/mL of *B*. *subtilis* 168 were inoculated into MRS broth, and cultivated aerobically for 96 h. Viable cell counts of *Lb*. *plantarum* SDMCC050204-pL157 were determined on MRS agar supplemented with chloramphenicol.

### Autolysis of *B. subtilis* in simulated intestinal fluid

*B*. *subtilis* 168 spores (2.0 × 10^7^ CFU/mL) were incubated in LB medium for 9 h to reach late logarithmic phase to early stationary phase. The vegetative cells were collected, washed and resuspended in simulated intestinal fluid (SIF) at an OD_600_ of 17.00 ± 0.46. The bacterial suspensions were incubated at 37 °C with agitation (200 rpm) to simulate peristalsis, and aliquots were taken to determine the OD_600_ at 0, 12 and 24 h. The SIF contained 0.5% NaCl, 1 g/L pancreatin (Sigma, USA), and 0%, 0.05%, 0.1% or 0.3% pig bile salts (Sigma, USA), pH 8.0^23^.

### Cultivation of *Lb*. *plantarum* with the autolysate of *B*. *subtilis* 168

To prepare autolysate of *B*. *subtilis* 168, vegetative cells were resuspended in 0.1% NaCl to one-tenth of the initial culture volume and stored at 4 °C for 7 days. Then, the intact cells and cell debris were removed by centrifugation at 13,000 × *g* for 20 min. The resultant supernatant was filtered with a 0.22 μm membrane and then heated at 100 °C for 15 min to inactivate any proteinic enzymes to obtain the autolysate. Culture medium was prepared by mixing an equal volume of two-fold concentrated MRS broth with the autolysate, or with 0.1% NaCl as a control. Then, 5.6 × 10^8^ CFU/mL *Lb*. *plantarum* SDMCC050204 were inoculated into the culture medium and incubated aerobically for 60 h.

### Analysis of catalase activity and H_2_O_2_ concentration

Cell pellets from 2 mL of *Lb*. *plantarum* SDMCC050204 culture were collected, washed three times with sterile saline solution, and resuspended in 50 μL saline solution. The cell suspensions were mixed with 20 μL of 30% H_2_O_2_ solution, and the air bubble formation was determined^24^. H_2_O_2_ concentrations in the culture were measured using a H_2_O_2_ Quantified Analysis Kit (Sangon Biotech, China) as stated by standard procedures.

### Detection of heme in the autolysate of *B*. *subtilis*

Heme was extracted from the autolysate of *B*. *subtilis* 168 according to the Weinstein method with some modifications^25^. Specifically, 100 mL of autolysate was concentrated to 10 mL by lyophilization (Thermo Savant, USA). Then, 30 mL of 90% aqueous acetone containing 5% HCl (v/v) were added to the autolysate. The mixture was vortexed for 10 min at room temperature, and then centrifuged at 13,000 × *g* for 10 min. The heme-containing supernatant was recovered, while the pellets were extracted with another 10 mL of acidic acetone. After centrifugation, the two fractions of supernatant were combined followed by evaporation in a vacuum rotary evaporator (Thermo Savant, USA) to remove the organic phase and a vacuum freeze-dryer to remove the water phase. The dry residue was dissolved in 3 mL of distilled water and the pH was adjusted to 12.0 for the transformation of heme to soluble hematin. The solution was filtered with a 0.22 μm membrane for the further detection.

Hematin was detected by high-performance liquid chromatography (HPLC; Shimadzu, Japan) using an XBridge BEH300 C18 reverse phase column (150 × 4.6 mm; Waters, USA) with a flow rate of 0.6 mL/min. The column was equilibrated with solvent A. Separation of the hematin was effected with a gradient of 20% to 70% solvent B over 40 min. The column effluent was monitored by photo-diode array detection at 398 nm. Solvent A was 0.1% trifluoroacetic acid (TFA) (v/v) in water; solvent B was 0.1% TFA (v/v) in acetonitrile. Hematin sample as prepared above (50 μL) was injected into the column for analysis. 10 μL of 30 μg/mL hematin (Sigma, USA) was used as the standard sample.

### Disruption of the heme-dependent catalase gene (*katA*) in *Lb*. *plantarum* SDMCC050204

Molecular cloning techniques were performed essentially as described previously^26^. *Taq* polymerase, restriction enzymes and T4 DNA ligase were used according to the manufacturer’s instructions (TaKaRa, Japan).

Disruption of the *katA* gene was carried out using a single-crossover integration strategy^27^. A DNA fragment containing the *katAk* was PCR amplified from *Lb*. *plantarum* SDMCC050204 genomic DNA with primers laX-1 (5′-ACCTCTAGAGACGTGCGCGGTTTT-3′; the underlined bases indicate an XbaI site) and laX-2 (5′-CAGAAGCTTAGGCTCGTAGTTGACC-3′; the underlined bases indicate a HindIII site). The PCR products were digested with XbaI/HindIII and then ligated into the compatible end of pUC-erm, yielding pUC-erm-kat. pUC-erm-kat (200 ng) was introduced into competent *Lb*. *plantarum* SDMCC050204 cells by electroporation. Transformants were selected on MRS agar containing erythromycin at a final concentration of 5 μg/mL, generating the mutant *Lb*. *plantarum* SDMCC050204ΔkatA.

### Evaluation of DNA damage due to H_2_O_2_ stress

Evaluation of DNA damage was conducted according to the methods of Rezaïki with some modifications^28^. Briefly, *Lb*. *plantarum* SDMCC050204 and *Lb*. *plantarum* SDMCC050204ΔkatA were incubated with or without the autolysate of *B*. *subtilis* 168 or heme for 12, 36 and 60 h. After cultivation, the genomic DNA was extracted using a TIANamp Bacteria DNA Kit (TIANGEN, China) and electrophoresed on Tris-acetate-EDTA agarose (1.0%) gels to compare the extent of DNA degradation in each sample.

### Statistical analysis

Statistical analysis was performed using data from three technical replicates by unpaired two-tailed Student’s *t*-test. *P* values of <0.05 were considered statistically significant; *P* values of <0.01 were considered statistically highly significant.

## Results

### Detection of survival of *Lb*. *plantarum* SDMCC050204-pL157 co-cultured with *B*. *subtilis* 168 *in vitro*

**Figure 1:**
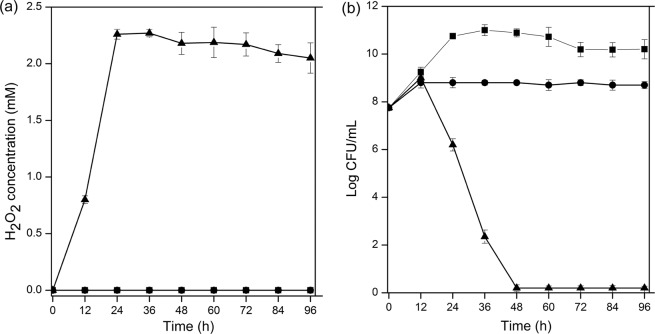
H_2_O_2_ accumulation (**a**) and viable cell counts (**b**) of *Lb*. *plantarum* SDMCC050204-pL157 when statically cultivated (■), and aerobically cultivated with (●) or without (▲) *B*. *subtilis* 168. In all panels, the values are means ± standard deviations of three independent experiments. The curves with symbols ■ and ● overlap in panel (a).

It was reported that oral administration of *Bacillus* favored the growth of *Lactobacillus* in the intestinal tract^29,30^. To confirm this result *in vitro*, *Lb*. *plantarum* SDMCC050204-pL157 was incubated with or without *B*. *subtilis* 168. The H_2_O_2_ concentrations and cell viability of *Lb*. *plantarum* SDMCC050204-pL157 were detected. As shown in Fig. 1a,b, when *Lb*. *plantarum* SDMCC050204-pL157 was cultivated alone under aerobic conditions for 96 h, about 2 mM H_2_O_2_ was detected in the cultures, and the viable cell numbers showed a significant decrease, from 5.62 ± 0.2 × 10^7^ CFU/mL at 0 h to 0 CFU/mL at 96 h. In contrast, in the static culture, very little H_2_O_2_ was detected, and the viable cell counts were 1.0 ± 0.4 × 10^9^ CFU/mL at 96 h, suggesting that *Lb*. *plantarum* SDMCC050204-pL157 suffered from H_2_O_2_ stress under aerobic conditions, causing cellular damage.

To test whether *B*. *subtilis* could decrease the H_2_O_2_ level in aerobic culture of *Lb*. *plantarum*, *Lb*. *plantarum* SDMCC050204-pL157 as well as two other *Lb*. *plantarum* strains SDMCC050276-pL157 and SDMCC050277-pL157 were respectively co-cultured with *B*. *subtilis* 168. The results showed that the H_2_O_2_ was not detectable in the co-cultures (Fig. 1a), as that of the static culture, and the viable cell counts of SDMCC050204-pL157 was 8.4 ± 0.1 × 10^8^ CFU/mL after 96 h incubation, close to that in the static culture (Fig. 1b). Similar phenomena were observed for co-cultivation of *B*. *subtilis* 168 with strains SDMCC050276-pL157 and SDMCC050277-pL157 (data not shown). These results confirmed that *B*. *subtilis* 168 could provide bioactive molecules to protect *Lb*. *plantarum* strains from H_2_O_2_ stress.

### Autolysis of *B*. *subtilis* 168 in SIF

**Figure 2:**
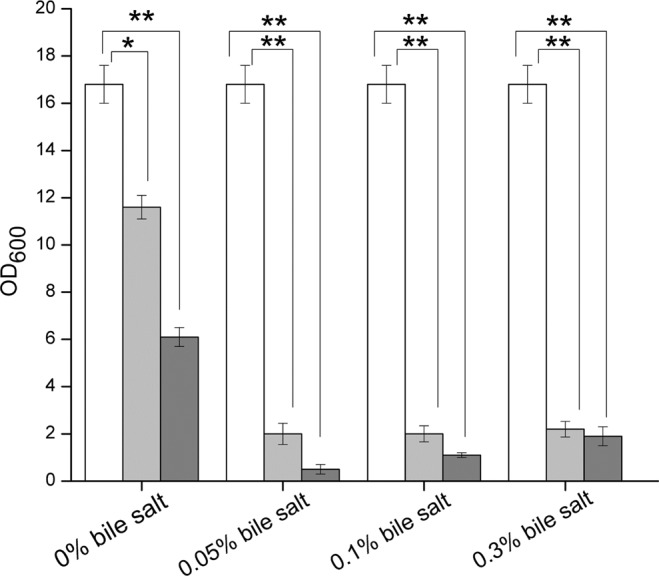
Autolysis of *B*. *subtilis* 168 cells in simulated intestinal fluid containing 0%, 0.05%, 0.1% and 0.3% pig bile salts determined by the decline of OD_600_ of cell suspensions. White column, 0 h incubation; light grey column, 12 h incubation; dark grey column, 24 h incubation. The values are means ± standard deviations of three independent experiments. *P < 0.05; **P < 0.01.

*B*. *subtilis* is prone to autolysis due to environmental stressors or the regulated processes that occur at different stages of the cell life^31,32^. To find out the bioactive molecules, *B*. *subtilis* 168 cells were suspended in SIF, and the OD_600_ was determined at 12 and 24 h, respectively. As shown in Fig. 2, autolysis of *B*. *subtilis* 168 cells was observed, and lysis extent reached up to 60% of the cells after 24 h. Moreover, the autolysis was significantly boosted up to 90% of the cells in the presence of 0.05% bile salts, revealing that *B*. *subtilis* 168 cells could autolyse in the intestinal tract.

### Activation of heme-dependent catalase by the autolysate

**Figure 3:**
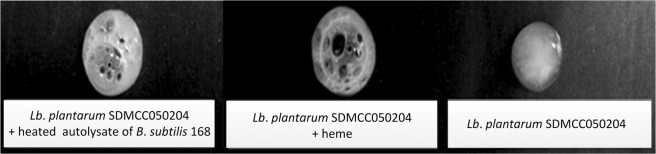
Qualitative analysis of catalase activity of *Lb*. *plantarum* SDMCC050204 in medium supplemented with the heated autolysate of *B*. *subtilis* 168, heme, or without supplementation. Catalase activity was indicated by bubble formation due to liberation of O_2_.

*B*. *subtilis* cells could autolyse, accompanied by the release of intracellular solutes. Here, solution containing the intracellular solutes of *B*. *subtilis* 168 without intact cells or cell debris was termed the autolysate. To identify the role(s) of the autolysate in the elimination of H_2_O_2_ from *Lb*. *plantarum* culture, the germfree filtering lysate of *B*. *subtilis* 168 cells was heated at 100 °C for 15 min to inactivate any proteinic enzymes, including catalase, peroxidase, superoxide dismutase, and so on. Then, *Lb*. *plantarum* SDMCC050204 was incubated in MRS medium supplemented with the heated autolysate for 24 h. As a result, H_2_O_2_ was undetectable in the cultures (data not shown), while air bubble formation was observed from cells of *Lb*. *plantarum* SDMCC050204 when treated with H_2_O_2_ (Fig. 3), suggesting that the heated autolysate contributed biomolecules that activated catalase activity in *Lb*. *plantarum* SDMCC050204. The same phenomenon was obtained when addition of exogenous heme to the medium instead of the heated autolysate. However, nor did these results when *Lb*. *plantarum* SDMCC050204 was cultivated in the medium without the heated autolysate or heme (Fig. 3). Thus, we concluded that the autolysate of *B*. *subtilis* 168 supplied heme, which in turn resulted in activity of the heme-dependent catalase KatA in *Lb*. *plantarum* SDMCC050204.

### Detection of heme in the autolysate of *B*. *subtilis* 168

**Figure 4:**
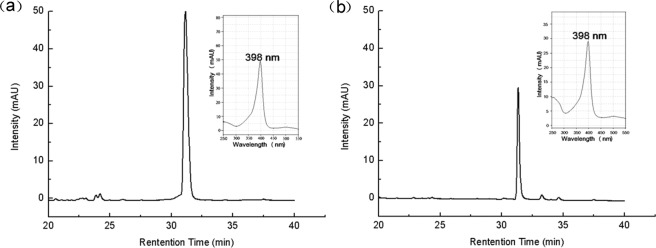
Detection of hematin in the autolysate of *B*. *subtilis* 168 by HPLC analysis. (**a**) Standard sample of hematin. (**b**) Sample extracted from the autolysate of *B*. *subtilis* 168. In the inset panels, the Soret band region of the spectrum of hematin is shown. Hematin is a soluble transforming product of heme.

To confirm the presence of heme in the autolysate, HPLC analysis was performed. As shown in Fig. 4, a specific absorption peak (retention time = 31.36 min) at 398 nm appeared, close to that for a standard hematin sample (retention time = 31.41 min), indicating that heme could be quantitatively provided by the autolysis of *B*. *subitlis* 168 cells.

### Functional analysis of heme-dependent catalase in *Lb*. *plantarum* SDMCC050204

**Figure 5:**
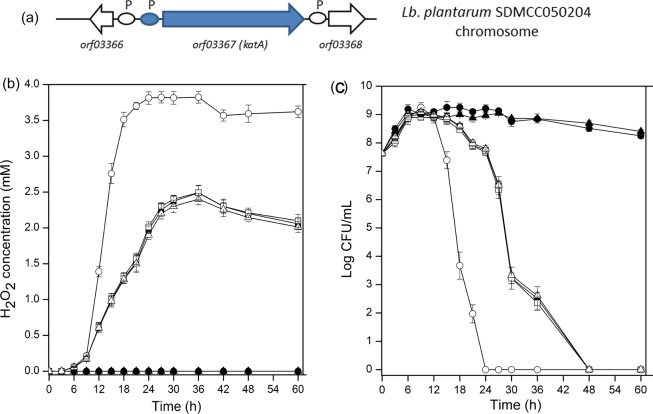
The genetic organization of katA in *Lb*. *plantarum* SDMCC050204 (**a**), H_2_O_2_ accumulation (**b**) and viable cell counts (**c**) of *Lb*. *plantarum* SDMCC050204 (solid symbols) or *Lb*. *plantarum* SDMCC050204ΔkatA mutant (open symbols) when cultivated aerobically alone (■, □), with the heated autolysate of *B*. *subtilis* 168 (●, ○), or with heme (▲, △). In panel (a), P indicates putative promoter. In panels (b,c) the values are means ± standard deviations of three independent experiments. The curves with symbols ■, □ and △ partially overlap.

Genomic sequencing indicated that a heme-dependent catalase coding gene, *katA*, is present in the genome of *Lb*. *plantarum* SDMCC050204 (unpublished data). Figure 5a shows the genetic organization of *katA* in *Lb*. *plantarum* SDMCC050204. To determine whether the positive response of H_2_O_2_ decomposition in cultures of *Lb*. *plantarum* supplemented with heme or B. subtilis 168 autolysate was catalyzed by KatA, the *katA* gene was disrupted. Cells of the mutant SDMCC050204ΔkatA could not degrade H_2_O_2_ to yield detectable air bubbles, even in the presence of either the autolysate of *B*. *subtilis* 168 or heme (data not shown).

The H_2_O_2_ concentrations in cultures of mutant SDMCC050204ΔkatA and the wild type SDMCC050204 were compared after supplementation with the autolysate or heme. As stated above, about 2 mM H_2_O_2_ was detected in the culture of strain SDMCC050204 in MRS medium, the same was observed in culture of the mutant SDMCC050204ΔkatA. When the autolysate was added, no H_2_O_2_ was detected in culture of strain SDMCC050204, while as much as 3.6 mM H_2_O_2_ was present in culture of the mutant SDMCC050204ΔkatA (Fig. 5b). Heme addition resulted in similar comparison of H_2_O_2_ levels in the cultures of the wild type and the mutant. Moreover, the mutant strain exhibited an earlier and sharper decline in viable cell numbers (Fig. 5c). These results indicated that SDMCC050204ΔkatA lost the ability to produce active catalase even in the presence of the *B*. *subtilis* autolysate or heme, highlighting the heme-dependent catalase as the key component mediating interaction between the *Bacillus* sp. and the *Lactobacillus*. No significant differences were observed between the addition of the autolysate and exogenous heme in respect to H_2_O_2_ concentration or cell viability (Fig. 5b,c).

### Detection of DNA damage under oxidative stress

**Figure 6:**
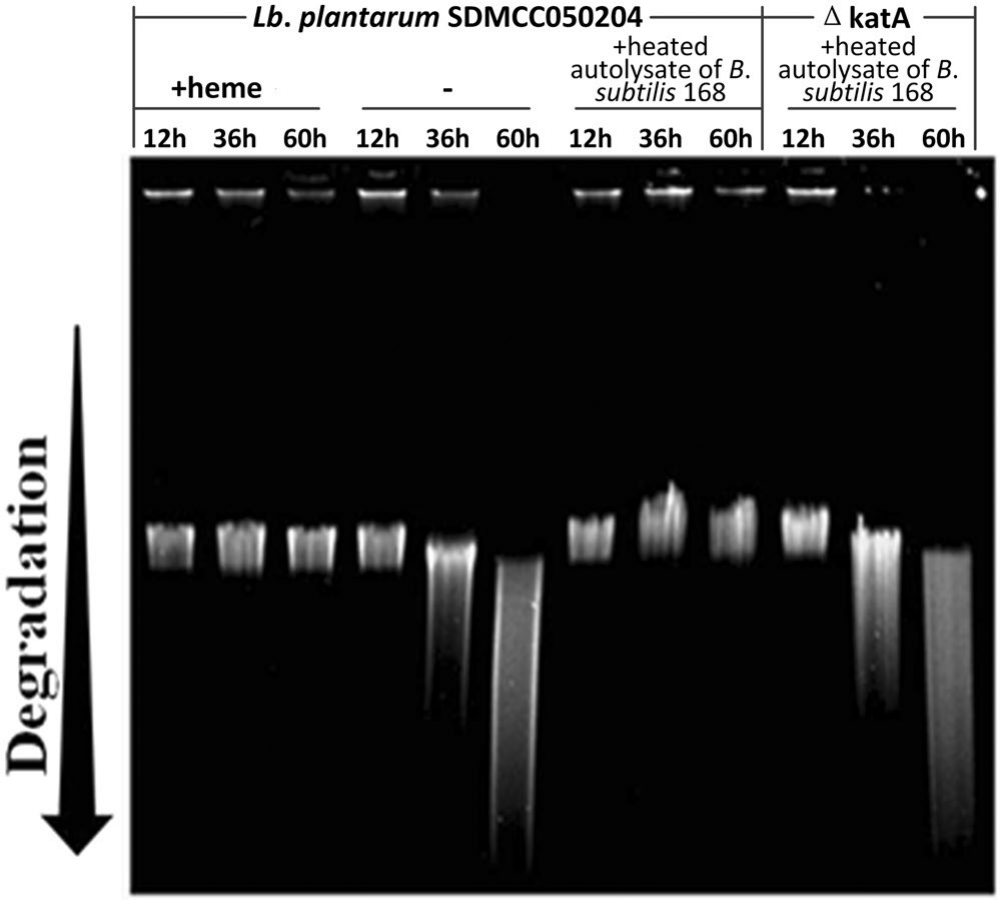
Evaluation of DNA damage of *Lb*. *plantarum* SDMCC050204 and the SDMCC050204ΔkatA mutant from aeration, aeration in the presence of heme, and aeration in the presence of the autolysate of *B*. *subtilis*. Chromosomal DNA was prepared from cells taken at 12, 36 and 60 h, and separated by electrophoresis on a 1% agarose gel to observe the extent of DNA degradation. Distance of migration reflects the degree of degradation, as indicated.

ROS have long been viewed as dangerous, highly-reactive molecules that cause cellular damage^17,28^. To examine DNA integrity, chromosomal DNA was extracted from cells of *Lb*. *plantarum* SDMCC050204 and SDMCC050204ΔkatA cultivated aerobically for 12, 36 and 60 h (Fig. 6). DNA damage was obviously alleviated in strain SDMCC050204 by the addition of heme or the *B*. *subtilis* 168 autolysate, but not in strain SDMCC050204ΔkatA. These results demonstrated that the activation of the heme-dependent catalase KatA efficiently protected *Lb*. *plantarum* strains against oxidative stress, and consequently helped maintain the DNA integrity.

## Discussion

It is generally considered that *Bacillus* strains are beneficial for the survival and growth of *Lactobacillus* in animal intestinal tracts on the basis of the “Biological Oxygen-Capturing Theory^9–12,33^”. According to this theory, the anaerobic environment generated by the growth of *Bacillus* strains plays major role in promotion of the growth of *Lactobacillus*^14^. Others have stated that catalase, subtilisin and surface proteins produced by *Bacillus* helped *Lactobacillus* inhabit the same niche^15,16^. In this study, we found that *B*. *subtilis* was prone to autolysis, particularly in the presence of bile salts, which offered the possibility to release heme that could be beneficial to other microbiota, including the core *Lactobacillus* in the intestinal tract. Our work focused on exploring the key factors linking the *B*. *subtilis* autolysate and *Lb*. *plantarum*, and the critical roles of heme and KatA, a heme-dependent catalase, were consequently demonstrated. This work thus proposed a novel crosstalk model between *Bacillus* and *Lactobacillus* in the intestinal tract, which would shed new light on the complicated interactions of different bacterial species in the gut microbiota.

ROS, including ${{\rm{O}}}_{2}^{-}$, H_2_O_2_ and ${\rm{H}}{\rm{O}}\cdot$, are generated as by-products of normal human cellular metabolic activities^34^. Alcohol, chronic infections and inflammatory disorders stimulate the production of ROS, and thus the intestinal tract is a key source of ROS^34,35^. Excessive accumulation of ROS results in oxidative stress, leading to intracellular biological macromolecular damage^34^. Meanwhile, the metabolism of *Lb*. *plantarum* is impacted by oxidative stress. H_2_O_2_ could induce the activity of pyruvate oxidase (POX), which converts pyruvate into acetate, accompanied by the production of extra H_2_O_2_^36^. Here, to imitate the ROS pool in the animal intestinal environment, aerobic cultivation was carried out to subject *Lb*. *plantarum* cells to oxidative stress, as 2 mM H_2_O_2_ was detected in the aerobic culture of *Lb*. *plantarum* SDMCC050204-pL157. We also found that *Lb*. *plantarum* was sensitive to oxidative damage from H_2_O_2_, agreeing with previous reports^37^. H_2_O_2_ damage to the *Lb*. *plantarum* cells mainly resulted from the lack of effective antioxidant systems. Although ROS erasers, including catalase, superoxide dismutase and NADH peroxidase, can be produced in some *Lactobacillus* strains by genetic and physiological analysis, the enzymatic activities are low^38,39^. In particular, catalase is commonly inactive in *Lactobacillus*, because the main cofactor heme is absent^18,40–42^. Therefore, mechanisms to cope with oxidative stress from H_2_O_2_ in the intestinal tract are of great importance for *Lactobacillus*. Our results here demonstrated that co-cultivation with *B*. *subtilis* significantly decreased the level of H_2_O_2_ and enhanced the survival of *Lb*. *plantarum* cells (Fig. 1).

*Bacillus* are complex organisms that exist as vegetative cells or metabolically inert spores or as part of a multicellular biofilm when encountering extreme environments^43^. When the environment is deficient in nutrients, rich in growth inhibitors and the pH or osmotic pressure is unfavorable, the vegetative cells of *Bacillus* are prone to autolysis, releasing resistant spores^31,32^. The above extreme conditions, to a large extent, can be provided by the gastrointestinal tract. Our results confirmed that most *B*. *subtilis* 168 vegetative cells spontaneously lysed in SIF (Fig. 2). The high-level autolysis released not only spores but also intercellular substances, making them candidate bioactive molecules to help *Lb*. *plantarum* resist H_2_O_2_ stress.

When exogenous heme or the autolysate of *B*. *subtilis* 168 devoid of catalase was added to the culture medium of *Lb*. *plantarum* SDMCC050204, the grown cell suspensions showed air bubble formation in the presence of H_2_O_2_. This result provided an indication of the presence of active catalase in cell suspensions of *Lb*. *plantarum*^25^. According to the genomic sequence, a heme-dependent catalase coding gene (*katA*) was in the genome of *Lb*. *plantarum* SDMCC050204. Thus, one hypothesis was that the autolysate of *B*. *subtilis* 168 offered heme to *Lb*. *plantarum* SDMCC050204 for activation of the heme-dependent catalase KatA. Then, heme was confirmed to be present in the autolysate of *B*. *subtilis* 168 (Fig. 4). Moreover, after disruption of the *katA* gene in the *Lb*. *plantarum* SDMCC050204 genome, the autolysate could not help *Lb*. *plantarum* SDMCC050204 to decrease the H_2_O_2_ concentration, avoid DNA damage and survive H_2_O_2_ stress (Figs 5 and 6). Thus, KatA and the heme provided by the autolysis of *B*. *subtilis*, were identified as important elements involved in the communication between *Lb*. *plantarum* and *B*. *subtilis*. The observation that strain SDMCC050204ΔkatA exhibited an earlier and sharper decline in viable cells than the wild type suggested that there were also other substances in the autolysate that promoted the growth of *Lb*. *plantarum* (Fig. 5c). In keeping with this speculation, small peaks in addition to the major one (hematin) were visible during HPLC analysis of the autolysate of *B*. *subtilis* 168. Our future work will focus on characterization of these unknown bioactive compounds produced by.

In conclusion, this study explored the molecular mechanisms by which *Lb*. *plantarum* benefits from co-culture with *B*. *subtilis*. It is suggested that the heme-dependent catalase KatA was activated, and this change significantly improved the survival of *Lb*. *plantarum* under H_2_O_2_ stress. Heme and KatA were the key linkage, whose functions were highlighted during the interaction of the two species. Furthermore, a crosstalk model in the intestinal tract was proposed: the life cycle of *Bacillus* strains led to the release of sufficient heme into the environment; the activity of *Lactobacillus* catalase was stimulated by the heme; H_2_O_2_ was degraded and oxidative stress was relieved; thus, the survival of *Lactobacillus* was promoted. Our finding will promote better and rational use of the two probiotics.
